# Resource utilization in lung cancer diagnostic procedures: Current use and budget consequences

**DOI:** 10.1371/journal.pone.0189251

**Published:** 2017-12-07

**Authors:** Sander Brinkhof, Harry J. M. Groen, Sabine S. Siesling, Maarten J. IJzerman

**Affiliations:** 1 Department of Health Technology and Services Research, University of Twente, Enschede, the Netherlands; 2 Department of Pulmonary Diseases, University Medical Center Groningen, University of Groningen, Groningen, the Netherlands; 3 Comprehensive Cancer Organisation, Utrecht, the Netherlands; 4 Department of Radiology, Medical School, University of Groningen, Groningen, the Netherlands; National Yang-Ming University, TAIWAN

## Abstract

**Objectives:**

The main objective of this study is to determine the current use of lung cancer diagnostic procedures in two large hospitals in the Netherlands, to explore deviations in guideline adherence between the hospitals, and to estimate the budget impact of the diagnostic work-up as well as the over- and underutilization.

**Materials & methods:**

A state transition model for the diagnostic pathway for lung cancer patients was developed using existing clinical practice guidelines (CPG) combined with a systematic literature. In addition to the CPGs depicting current practice, diagnostic utilization was gathered in two large hospitals representing an academic tertiary care hospital and a large regional teaching hospital for patients, who were selected from the Netherlands cancer registry.

**Results:**

The total population consisted of 376 patients with lung cancer. Not in all cases the guideline was followed, for instance in the usage of MR brain with stage III lung cancer patients (n = 70). The state-transition model predicts an average budget impact for the diagnostic pathway per patient estimated of € 2496 in the academic tertiary care hospital and € 2191 in the large regional teaching hospital.

**Conclusion:**

The adherence to the CPG’s differed between hospitals, which questions the adherence to CPG’s in general. Adherence to CPG’s could lead to less costs in the diagnostic pathway for lung cancer patients.

## Introduction

Lung cancer is characterized as one of the most malignant tumors with significant mortality and a large health and economic impact. The incidence in western countries is about 0.04 percent, which is around 12 percent of all new cancer diagnoses [1]. Lung cancer has a bad prognosis with an average survival rate of fifteen percent in five years [2]. The prognosis is often worse, because a majority of cases present with advanced stage disease—when treatments can rarely achieve cure[3,4].

Several countries have implemented lung cancer diagnostic and treatment guidelines. The diagnostic guidelines can support the development of fast and efficient care pathways, which are required, because a fast and early diagnosis can lead to a better prognosis [5,6]. Roughly, the pathway can be divided into three parts: imaging, invasive examination and functional examination. Imaging includes X-ray, CT and PET/CT and invasive examination; acquiring tumor tissue via endoscopic ultrasound (EUS), endobronchial ultrasound (EBUS), cervical mediastinoscopy or a CT guided biopsy.

Several changes in lung cancer diagnostics are currently investigated, including early detection by means of population based screening of high-risk patients. Moreover, the influence of demographic changes such as an ageing population, and the implementation of mutation profiling such as EGFR and ALK testing will have impact on the need for and amount of diagnostic procedures. These changes will influence the required resources, hence the care pathway is challenged for efficiency.

As an illustration, several countries are working toward implementation of early detection by means of screening. Yet, screening programs are known for their down-stream effects on hospital resources, because patients are submitted for additional diagnostic procedures which allow identification of tumors and possible metastases, both by physical examination as well as via biopsy [7]. In addition, several high-sensitive technologies are being developed to detect early stage malignancies, such as the detection of methylated DNA [8,9], cell-free DNA [10] or Circulating Tumour Cells [11,12]. Such technologies will impact the current health system, both in terms of early detection, hopefully leading to an increased survival, as well as an expected increase in the number of patients submitted for further diagnostic procedures and potentially treatment.

The main objective of this study is to determine the current use of diagnostic procedures in two large hospitals, to explore guideline adherence, differences between these hospitals and to estimate the budget impact of the diagnostic work-up as well as the over- and underutilization of lung cancer diagnostic procedures.

## Methods

### Diagnostic pathway

As the basis for the transition model the diagnostic pathway was defined and expressed in a flowchart, based on the clinical practice guidelines (CPG). This study assumes the clinical practice guidelines for lung cancer do represent current diagnostic practice. Experts, such as chest physicians and nurse practitioners, are consulted in addition to these CPG’s to see whether these guidelines are used in practice and which deviations occur. The information gained from CPG’s is combined with results from a systematic literature search for diagnostic procedure models to create an overview for the model.

### Current diagnostic practice

In addition to the CPGs depicting current practice, two large hospitals representing an academic tertiary care hospital and a large regional teaching hospital were selected to collect diagnostic resource utilization. Patients diagnosed in 2012 with lung cancer were selected from the Netherlands Cancer Registry (NCR). The NCR collects data on patient, tumor (i.e stage according to the TNM system[13], treatment and vital status of all malignancies in the Netherlands based on pathology notification and hospital discharge registries[2]. Detailed data on diagnostics were gathered directly from the patient files in the two participating hospitals. Every patient was followed through the diagnostic pathway to see what modalities were used in practice.

This one-year cohort is assumed to be representative for the in-hospital diagnostic procedures in lung cancer, and is used in the state-transition model to generalize to prospective patients.

### State transition model

For the development of the state transition model frequencies of use of diagnostic modalities were determined, using patient level data. These are combined in a model, in which states are based on investigations or tests that lead to the stages of the TNM staging system[13]. The TNM staging system consists of the presence of a tumor (T), occurrence of lymph node involvement (N) and existence of metastases (M). The starting point in the model is the suspicion for lung cancer. The overall endpoint is the TNM stage of the tumor, which can also be the absence of malignancies. This state-transition model [14] of the diagnostic pathway is developed and built in Microsoft Excel (Redmond, Washington, 2010). The model uses tariffs from Dutch Healthcare Authority (NZa), which are acquired via the NZa cost application.

### Statistical analysis

A beta distribution is used for the usage rates in the model due to the binomial nature of these parameters. A chi-squared test is used in SPSS (IBM Corp., Armonk, NY, 2013) to assess independence of the usage rates in both hospitals.

## Results

The total pathway for patients with suspected lung cancer which is modeled consists of 20 different modalities. A population consisting of 376 consecutive patients with lung cancer selected in two hospitals (two large hospitals representing an academic tertiary care hospital and a large regional teaching hospital) is used to assess the diagnostic pathway of lung cancer diagnosis. Table 1 gives an overview of both the populations in both hospitals representing a total of 376 patients, with a mean age of 67 and 65,8 respectively.

**Table 1:** Population characteristics.

Population characteristics*	Large teaching hospital (n = 214)	Academic tertiary hospital (n = 162)
Mean age (se)	66.95 (10.335)	65.77 (10.180)
Gender (male)	136 (63.6%)	97 (59.9%)
Small cell carcinoma Non-small cell carcinoma - Adenocarcinoma - Squamous cell carcinoma - Large cell carcinoma - Other	28 (13.1%) 186 (86.9%) 89 (41.6%) 59 (27.6%) 3 (1.4%) 35 (16.4%)	29 (17.9%) 133 (82.1%) 64 (39.5%) 42 (25.9%) 11 (6.8%) 16 (9.9%)

* Cohort with lung cancer diagnoses in 2012

The number and frequency (usage rates) of the diagnostic procedures are listed in Table 2. The usage rates are depicted as absolute (times used) and relative (percentage of total population). Significant differences are seen in the diagnosis for the M parameter, such as the PET-CT (p = 0.008), the diagnostic CT (p = 0.011) and the MRI of the brain (p = 0.005), the latter is more often performed at the University Medical Center. This approach provided a better risk estimation in case of surgery for early and oligometastatic patients.

**Table 2:** Usage of diagnostic procedures in diagnostic pathway.

Modality	Large teaching hospital	Usage rate (%)	Academic tertiary hospital	Usage rate (%)	p-value
X-thorax	186	87.7	131	86.1	0.241
PET/CT	155	73.1	133	87.5	**0.008**
CT	209	98.5	147	96.7	**0.0111**
Cervical mediastinoscopy	12	5.66	9	5.92	0.976
EUS/EBUS	52	24.5	46	30.2	0.305
MR brain	37	17.5	47	30.9	**0.005**
Bone scintigraphy	9	4.24	2	1.31	0.097
Thoracocentesis	28	13.2	21	13.8	0.962
Bronchoscopy	142	67.0	101	66.5	0.596
X-ray guidance bronchoscopy	33	15.6	24	15.8	0.941
CT guided biopsy	25	11.8	31	20.3	**0.035**
X-thorax after biopsy	23	10.8	13	8.55	0.411
VO2 max	23	10.8	17	11.2	0.995
Broad lung function	137	64.6	68	44.7	**<0.001**
Flow volume	1	0.47	34	22.4	**<0.001**
Ventilation / perfusion scan	19	8.96	11	7.24	0.497
ECG	98	46.2	125	82.2	**<0.001**
KRAS / EGFR	64	30.2	41	27.0	0.391
ALK	8	3.77	15	8.55	**0.023**

The tariffs for each diagnostic procedure are listed in Table 3. Using these tariffs for the state-transition model leads to an average budget impact for the diagnostic pathway per patient estimated at € 2496 in the academic tertiary care hospital and € 2191 in the large regional teaching hospital. These differences are explained by the fact that novel diagnostic modalities such as mutation profiling, which are carried out more by the academic hospitals.

**Table 3:** Procedure and test tariffs (2014).

Modality	DBC procedure code	Tariff (€)	Hospital costs (€)
X-thorax	85002	43.66	36.19
CT-thorax	85042	182.21	132.19
PET/CT	120501	1162.93	951.29
EUS/EBUS	34386/34387	791.15	622.59
Mediastinoscopy	29099045	2430.00	2430.00
Brain MR	81092	218.29	173.21
Thoracocentesis	32684	50.50	50.50
Bronchoscopy	32480	370.71	370.71
X-ray guidance	85000	53.14	48.66
CT-guided biopsy	80047	229.63	162.42
VO2 max	39844	170.38	88.13
Flow volume + diffusion	39932	67.60	67.60
Flow volume	39839	33.11	33.11
Ventilation/perfusion scan	120060	225.78	165.31
ECG	39757	26.83	26.83
Mutation analysis KRAS/EGFR	50512	951.54	905.18
Translocation analysis ALK	50514	449.30	393.57

Note: DBC procedure code are the codes used in the Netherlands for reimbursement.

Differences with guideline adherence are especially seen for the number of MRI brains used, which is different between both hospitals and not according to the guidelines. With this casemix, the academic center should have had 27 MRI brains (16.7% of casemix with stage III tumor), while 47 (21.4%) were actually carried out. Within the tertiary center, this difference is smaller: 43 according to guidelines (20%), 37 (17.2%) actually carried out. Lastly, a PET/CT is carried out more often in the academic hospital (p = 0.008), which can be explained by the fact that the teaching hospital has to refer patients to another hospital for a PET/CT.

## Discussion & recommendations

This study summarizes resource use in two large hospitals in the Netherlands, representing an academic tertiary care hospital and a large regional teaching hospital. The datasets consist of 376 patients, which represent all patients diagnosed with lung cancer in 2012 (162 and 214 respectively). The average costs per patient for the diagnostic pathways ranged between €2200 in the large regional teaching hospital and €2500 in the academic tertiary care hospital, which ends up to at least ten percent of the yearly costs of lung cancer treatment[15,16].

Two populations are studied from two different hospitals, one large academic center (162 of 407 patients in the province) and one large regional teaching hospital (214 of 713 patients in the province) [17]. This gives insight and could not be generalized to all other hospitals but to those who have a situation alike. A large difference in adherence to CPG’s in these two centers can be seen with the usage rates for PET/CT, brain MRI and advances in novel tests such as mutation analysis (detection of ALK translocations in tumor tissue). The deviation in usage rate for PET/CT can be explained by the fact that the regional hospital had to refer patients to a different location for the PET/CT. This difference in availability between centers could lead to a cost difference.

Deviations can also occur due to usage of more diagnostic procedures than sometimes necessary, which can be seen in different usage rates in PET/CT, diagnostic CT, CT guided biopsy, cardiac and lung function tests. In general, about 18 tests are performed in individual patient to diagnose and have functional information whether procedures, surgery, targeted therapy or chemotherapy can be performed. But it also means that even with CPG’s there is practice variability due to variability in patients characteristics.

A limitation in our data is the fact that not all diagnostics carried out for referred patients to the academic center are listed, because we did not have access to the files at the original hospital. Patients are selected through the dataset of the Netherlands Cancer Registry, hosted by the Netherlands Comprehensive Cancer Organisation, which registers all patients whom are diagnosed with lung cancer in the year 2012 in both hospitals. However, these patients represent the true positives, i.e. those with confirmed lung cancer. Patients suspicious of lung cancer who finally appear false-positive cannot be identified through the cancer registry. However, it should be acknowledged that this group is an important group to calculate the overall budget impact of the lung cancer diagnostic pathway. As mentioned above, a dataset of patients with lung cancer in 2012 is used. The Dutch guidelines did not change since 2011 apart from a minor addition in the detection of oncogenic abnormalities. The authors therefore chose this dataset, because full data access was granted.

Due to the increasing age of the population and the increase in incidence of lung cancer, it is expected the need for diagnostic procedures will dramatically impact the health system. Hence, this will result to an increase in resources and thus higher budget impact. For that reason, there is an increased need for a sustainable diagnostic pathway for suspected lung cancer patients.

The adherence to the CPG’s differ in the two hospitals, which questions the adherence to CPG’s in general. Guideline adherence in the diagnostic workup has been questioned before in other settings, for instance the use of an MRI prior to breast surgery. This study showed considerable hospital variation in the use of a breast MRI (range 4–84%)[18]. This has also been shown in a large cohort study of 15.951 lung cancer patients, of which 40% did not receive adequate guideline-concordant care[19].

Overuse or misuse of diagnostic modalities can lead to a high burden on the budget impact. Adherence to CPG’s could lead to less costs in the diagnostic pathway for lung cancer patients. A more thorough evaluation into all applied tests is needed in a larger population to have more accurate data on costs and their regional variation.
